# Sex differences in prevalence of metabolic syndrome components and contributing factors among psychiatric patients in Saudi Arabia

**DOI:** 10.3389/fpubh.2025.1579145

**Published:** 2025-04-14

**Authors:** Ali Abdullah Alyousef, Pandurangan Subash-Babu, Ghedeir M. Alshammari, Kholoud B. Alabdulkareem, Abu ElGasim A. Yagoub, Mohammed Abdullah Alomar, Abdulmohsen jasem Alrashed, Mohammed A. Mohammed, Mohammed Abdo Yahya

**Affiliations:** ^1^Department of Food Science and Nutrition, College of Food and Agricultural Science, King Saud University, Riyadh, Saudi Arabia; ^2^Department of Social Studies, College of Arts, King Saud University, Riyadh, Saudi Arabia; ^3^Mental Health Hospital, Al-Ahsa, Ministry of Health, Hofuf, Saudi Arabia

**Keywords:** metabolic syndrome, adult, gender, dietary habits, risk factors

## Abstract

**Background/objectives:**

The purpose of this study was to detect sex differences in metabolic syndrome (MS) features and risk variables among Saudi participants.

**Methods:**

For this study, 144 patients (52.08% males, 47.92% females) aged 19 to 59 signed a written consent form based on the Declaration of Helsinki, either as a patient or a family member. A cross-sectional survey was used to collect data on family disease history, health, and eating habits. MS components included waist circumstance (WC), high-density lipoprotein cholesterol (HDL-C), triglycerides (TGs), fasting glucose (FG), and blood pressure. A chi-square test was used to compare categorical data within and across sexes.

**Results:**

The associations between variables were found using Spearman correlation coefficients and simple regression analysis. Anthropometric indices were significantly (*p* ≤ 0.01) varied between sexes as well as family history, health behaviors, and eating behaviors. Sex variations in MS components that contributed to an MS diagnosis were discovered and were significantly varied between sexes. The most common components in males were low HDL-C (90.67%), high WC (85.33), and elevated TG levels (76.0%). In females, the most typically recognized components were high WC (86.96%), followed by high FG (69.57%) and high blood pressure and TG (63.77%). Sex differences in other risk variables for MS, such as family history, health, dietary habits, sedentary lifestyle, and smoking, were found to be associated with high anthropometric indices.

**Conclusion:**

Sex-specific public health policies and management techniques for preventing MS in the older adult population should be created for Saudis who are aging physiologically.

## Introduction

Metabolic syndrome (MS) is a multifactorial risk factor for cardiovascular disease, type 2 diabetes, and other health consequences that poses a significant challenge to both clinical practice and public health. The rising world's incidence of metabolic syndrome, caused by growing urbanization, sedentary lifestyles, and dietary changes, emphasizes the critical need to manage this condition (1). Diagnostic techniques vary, but they often focus on abdominal obesity (measured by waist circumference), hyperglycemia, dyslipidemia, and hypertension, emphasizing the importance of population- and phenotype-specific diagnostic strategies. The care of metabolic syndrome promotes lifestyle changes such as healthy eating habits, physical activity, and excess visceral and extra uterine adiposity control as essential interventions (1). MS has been expanding globally for decades, particularly in underdeveloped nations (2). MS is diagnosed when there is abdominal obesity combined with two or more risk factors, such as high fasting triglycerides (TGs), poor high-density lipoprotein (HDL) cholesterol, raised blood pressure (BP), and high fasting plasma glucose (FPG) (3). A study found that MS prevalence varies by sex, age, and nation (4), and it is one of the most common diseases among the older adult (5). MS prevalence varies among older persons, with females having a higher incidence than males (6). According to Yi and An (7), the consequences of MS risk variables by sex and age are as follows: middle-aged persons, regardless of sex, face more risk factors for MS than any other age group. Thus, the suggested risk category for managing MS is middle-aged males and females. MS has been found to occur at a young age, and its prevalence is increasing globally as obesity rates rise (8). It is distinguished by the presence of activated macrophages and crown-like structures in adipose tissue, resulting in tissue injury (9). It is the result of a complex interplay of genetic, environmental, and lifestyle factors such as high-calorie intake and low levels of physical exercise (10). These variables cause insulin resistance, chronic inflammation, and neuro-hormonal alterations, all of which contribute to the progression and transition of MS to cardiovascular disease and diabetes (11). MS has been linked to external environmental factors such as demographics, socioeconomic status, health behavior, and lifestyle (12). Despite the development of a national health policy for MS, it is difficult to manage the risk factors associated with this condition during adulthood since health behavior and lifestyle are shaped by the interaction of genetic, social, and environmental variables beginning in childhood. Furthermore, variations among social groups create various hurdles to lifestyle adjustments (13). Previous investigations on the link between MS and depression showed contradictory findings. A study found no significant association between the two conditions (14). In contrast, another study found that the burden of metabolic syndrome is very high, particularly among patients with mental illness, compared to the general population, with a significant association between mental illness and metabolic syndrome (15). Saudi Arabia has made enormous socioeconomic progress as a result of developments in health, education, and the environment, which have led to rural-urban migration and lifestyle changes. Consequently, chronic disorders, including diabetes, obesity, and heart disease, are becoming more common. The prevalence of MS in Saudi Arabia has been estimated at 31.6% (IDF criteria) and 39.9% (ATP III criteria) (16). A cross-sectional study found that around 34.4% of males in Saudi Arabia have MS, with the percentage increasing with age, and that the most common MS components were low HDL-C and abdominal obesity (17). According to Khyzer and Aftab (18), the prevalence of MS among medical undergraduates in Arar, Saudi Arabia, is 21.5%. MS is more common among obese and female students with a strong family history of obesity and hypertension than in non-obese students. A study in the United Arab Emirates found a prevalence of 50.3% in 2012, which is greater than Jordan's rate (19). Another study conducted in Jordan indicated a prevalence of 41.7%, which is roughly comparable to the rates found in other studies (20). The significant disparity in MS prevalence across and between sexes, nations, and communities could be attributed to a combination of genetics, environmental features, variables, epidemiological transition, and lifestyle variations. Individual variation in metabolic syndrome is increased by both sex and age factors. Sex- and age-related factors of metabolic syndrome are sensitive to biological, environmental, and psychosocial conditions. Age differences cause sex differences in risk factors for metabolic syndrome; therefore, sex differences in metabolic syndrome prevalence are due to physiological differences such as hormones, differences in social and psychological stressors, and differences in lifestyle (7, 21). The goal of this study was to detect sex differences in prevalence of metabolic syndrome components and contributing factors among psychiatric patients in Saudi Arabia.

## Materials and methods

### subjects and sample size

A cross-sectional investigation was carried out in a Mental Health Hospital in Al-Has city, Saudi Arabia, between January and April 2022. The researcher (nutritionist) interviewed participants to check their eligibility for the study. A total of 144 eligible male and female Saudi patients were approached and accepted to participate in the study. Their ages ranged from 19 to 59, the number of male participants (52.08%) exceeded that of females (47.92%), and the majority of both were single, illiterate, and with low income. According to policies of the Saudi Ministry of Health, hospitalized patients are given a high-protein, high-calorie diet, which is fit for psychiatric patients. They are also provided with special diets such as low-fat and low-salt diets and other diets suitable for chronic diseases. The cross-section study's accuracy and prevalence of satisfaction were considered while calculating the sample size, and participants were chosen using inclusion and exclusion criteria. The inclusion criteria included patients of both sexes, over 18 years of age and older, and hospitalized MS patients, while the exclusion criteria included the patient's refusal to provide all relevant information, refusal to provide written informed permission to participate in the study from the participant or their legal representative and taking cognitive or psychotropic medications that may cause deterioration in the patient's health, preventing the collection of relevant information and the participant's ability to participate in the study fully. Pregnant or breastfeeding women were excluded.

To detect such a median-sized main effect with a statistical power of 1 – β = 0.80 (i.e., if the effect exists, there is an 80% chance of detecting a true positive) using a two-tailed test with an α of 0.05 (i.e., if the effect does not exist, there is a 5% risk of detecting a false positive). To calculate the required N, you enter the three key parameters (expected d, target power, α) in G^*^Power, which uses a formula (22):


$$\begin{array}{l} N=\frac{4\times \left(\;Z_{1-\alpha /2\;=0.025}+Z_{1-\beta \;=0.80}\right)2}{d_{\left(condition\right)2\;}} \end{array}$$

Where Z_(1−α/2 = 0.025)_ = 1.96 and Z_(1−β = 0.80)_ = 0.84 are the critical Z values associated with a two-tailed test with α = 0.05 and 1 – β = 0.80, *d* = 0.50 respectively. Simply put, assuming that your intervention works as expected, a sample size of 125 participants will give you an 80% probability to observe a median-sized (or larger) effect of Condition with *p* < 0.05.

### Data collection

A structured and validated questionnaire was approved by a nutritional expert committee at both King Saud University and Mental Health Hospital in Al-Has city, Saudi Arabia. The questionnaire involved questions concerning health behaviors, food habits, physical activity, smoking, and family history of disease were used to guide the structured interviews that were used to gather the data. The researchers and two assistant data collectors, who received the necessary training and became familiar with the purpose and goals of the study, designed and carried out the questionnaire. In-person interviews were used to choose study participants.

### Anthropometric indices

The anthropometric parameters were measured using an AC-CUNIQ BC360 (SELVAS Healthcare Inc., Daejeon, Republic of Korea) body composition analyzer validated and used according to manual instructions. Patients were asked to stand while electrodes were placed on their hands and feet to obtain measurements. Body mass index (BMI), waist circumference (WC), height, and body weight were automatically calculated when the device was connected to an ultrasonic height meter. Following each measurement, the patients' palms and soles were cleaned, and they were told to fast for 4 h before the test. The participants were given appropriate privacy. BMI and waist circumference were classified according to bioelectrical impedance analysis instruments (23). The participants' BF and VF were classified according to Kyle et al. (24) and by taking into consideration the age of respondents, whereas waist-to-height ratio (WHtR) was classified according to Selçuk (25).

### Blood sample collection and analysis

Each individual had a venous blood sample (10 mL) taken before the first meal. Within an hour after collection, the aliquot was centrifuged for 15 min at 3,000 rpm, and the serum was coded and stored at −80°C until biochemical analysis. An XN1000-Cobas E411 (Sysmex-Rauch-Hitachi, Tokyo, Japan) was used in the Mental Health Hospital's laboratories to detect high-density lipoprotein cholesterol (HDL-C), triglycerides (TGs), and fasting blood sugar (BS). Participants' blood pressures were also measured.

### Metabolic syndrome diagnosis

Participants were assessed based on their blood samples to see if they satisfied the MS criteria outlined in the National Cholesterol Education Program: Adult Treatment Panel III (NCEP: ATP III). According to the definition, participants were judged to have MS if they met at least three of five criteria, as stated by Cornier et al. (26). The classification criteria were waist-to-height ratio (WHtR), high-density lipoprotein cholesterol (HDL-C), triglycerides (TGs), fasting glucose (FG), and blood pressure (BP). Participants were judged to have MS (NCEP ATP111; 2008 revision) if they showed three of the following: individuals may have abdominal obesity (WC >90 cm for males or >80 cm for females), hypertriglyceridemia (≥150 mg/dL), low HDL-C (HDL-C < 40 mg/dL for males or < 50 mg/dL for females), high blood pressure (systolic blood pressure ≥ 130 mmHg or diastolic blood pressure ≥ 85 mmHg), and high fasting glucose (≥100 mg/dL).

### Ethical approval

The research protocol was approved by the Research and Ethical Committee of Academic Affairs and Research at King Fahad Hospital Hofuf (37-44-2021), committee meeting No. 44 (13 October 2021), and the work was carried out under the legal requirements and guidelines for good clinical practice.

### Statistical analysis

The data were analyzed with the statistical software SPSS (version 28.1; SPSS Inc., Chicago, IL, USA). The results were provided as frequencies and percentages. A chi-square test was used to determine the link between patients' variables. The statistical significance level was set to ^**^p ≤ 0.01 and ^*^p ≤ 0.05. The associations between family history, health, and dietary behavior, as well as MS components and anthropometric proxies, were investigated using Spearman correlation coefficients and simple regression analysis.

## Results

### Anthropometric indices of participants

Table 1 shows the anthropometric features of males and females with MS. The anthropometric proxies of male and female respondents with MS differed between sexes. According to BMI, males (40%) were significantly (*p* ≤ 0.01) more overweight than females (28.99%). Females with obesity (52.17%) had a significantly higher prevalence percentage (*p* ≤ 0.01) than males (42.67%). The waist-to-height ratio (WHtR) values were comparable to those obtained for BMI. The body fat (BF) study revealed that males (72%) had considerably (*p* ≤ 0.01) higher body fat levels than females (40.57%). Moreover, males had significantly (*p* ≤ 0.01) higher visceral fat (34.67%) than females (18.84%).

**Table 1:** Anthropometric indices of metabolic syndrome subjects with metabolic syndrome categorized by sex.

**Anthropometric**	**Males (*****n*** = **75)**		**Males chi-square**	**Females (*****n*** = **69)**		**Females chi-square**	**Sex chi-square**
	***F***	**%**		***F***	**%**		
**Body mass index (BMI)**						
Underweight	1	1.33	56.12^**^	4	5.80	27.96^**^	79.92^**^
Normal	12	16.0		9	13.04		
Overweight	30	40.0		20	28.99		
Obese	**32**	42.67		36	52.17		
Total	**75**	100.0		69	100.0		
**Waist-to-height ratio (WHtR)**						
Normal	9	12.0	11.88^**^	7	10.15	47.81^**^	50.72^**^
Overweight	18	24.0		9	13.04		
High overweight	18	24.0		11	15.94		
Obese	30	40.0		42	60.87		
Total	75	100.0		69	100.0		
**Body fat (BF)**						
Decreased	0	0.0	52.08^**^	7	10.15	13.26^**^	86.50^**^
Normal	6	8.0		19	27.54		
High	15	20.0		15	21.74		
Very high	54	72.0		28	40.57		
Total	75	100.0		69	100.0		
**Visceral fat (VF)**						
Decreased	1	1.33	23.51^**^	2	2.90	31.23^**^	43.89^**^
Normal	21	28.0		34	49.27		
High	27	36.0		20	28.99		
Very high	26	34.67		13	18.84		
Total	75	100.0		69	100.0		

^**^p ≤ 0.01.

### Family history, health, and eating behavior of participants

Table 2 displays the respondents' family histories as well as health and dietary behavior. Males and females differed significantly in most variables. A family history of diabetes, acute myocardial infarction, or cerebral infarction differed considerably between sexes, with males having 69.33% and females having 79.71% (*p* ≤ 0.01). The bulk of health-related conditions varied significantly between sexes. Females had a considerably higher percentage of elevated blood pressure (76.81%) than males (54.67%). Females (52.17%) exhibited significantly greater rates of elevated blood glucose or sugar in the urine (*p* ≤ 0.05) compared to males (38.67%). More than half of the males (54.67%) smoked, whereas 4.35% of the females did. Eating behaviors such as overeating eating did not differ significantly between sexes, but quick eating was significantly prevailing among males. Females were shown to be considerably (*p* ≤ 0.01) more likely than males to skip meals, as well as preferring salt or strong food flavors (*p* ≤ 0.01). Furthermore, the percentage of females (91.30%) who did not engage in physical activity was significantly lower (*p* ≤ 0.01) than that of males (93.33%).

**Table 2:** Family history, health behaviors, and eating behaviors of patients by sex.

**Variables**	**Males**		**Males *p*-value**	**Females**		**Females *p*-value**	**Sex** ***p*****-value**		
	***F***	**%**		***F***	**%**				
**Family (immediate or extended) diagnosis of diabetes, acute myocardial infarction, or cerebral infarction**									
Yes	52	69.33	11.21^**^	55	79.71	24.36^**^	107	74.31	34.03^**^
No	23	30.67		14	20.29		37	25.69	
Total	75	100.0		69	100.0		144	100.0	
**High blood pressure**									
Yes	41	54.67	0.653	53	76.81	67.57^**^	94	65.28	13.44^**^
No	34	45.33		16	23.19		50	34.72	
Total	75	100.0		69	100.0		144	100.0	
**High blood glucose or sugar in the urine**									
Yes	29	38.67	3.85^*^	36	52.17	0.72^**^	65	45.14	1.36
No	46	61.33		33	47.83		79	54.86	
Total	75	100.0		69	100.0		144	100.0	
**Smoking status**									
Yes	41	54.67	0.653	3	4.35	57.52^**^	44	30.56	21.78^**^
No	34	45.33		66	95.65		100	69.44	
Total	75	100.0		69	100.0		144	100.0	
**Eat excess food**									
Yes	34	45.33	0.653	34	49.28	0.014	68	47.22	0.44
No	41	54.67		35	50.72		67	52.78	
Total	75	100.0		69	100.0		144	100.0	
**Eating behavior (fast)**									
Yes	35	46.67	0.333	31	44.93	0.710	66	45.83	1.00^*^
No	40	53.33		38	55.07		78	54.17	
Total	75	100.0		69	100.0		144	100.0	
**Skipping meal**									
Yes	12	16.0	34.68^**^	20	28.99	12.19^**^	32	22.22	44.44^**^
No	63	84.0		49	71.01		112	77.78	
Total	75	100.0		69	100.0		144	100.0	
**Intake of salty foods**									
Yes	21	28.0	14.52^**^	26	37.68	4.19^*^	47	32.64	17.36^**^
No	54	72.0		43	62.32		97	67.36	
Total	75	100.0		69	100.0		144	100.0	
**Enjoying strong tasty foods**									
Yes	24	32.0	9.72^*^	18	26.09	15.78^**^	42	29.17	25.00^**^
No	51	68.0		51	73.91		102	70.83	
Total	75	100.0		69	100.0		144	100.0	
**Physical activity**									
Yes	5	6.67	56.33^**^	6	8.70	47.09^**^	11	7.64	103.36^**^
No	70	93.33		63	91.30		133	92.36	
Total	75	100.0		69	100.0		144	100.0	

*F*, frequency; ^**^p ≤ 0.01, ^*^p ≤ 0.05; diagnosis is based on the International Classification of Diseases and Related Health Problems (ICD-10).

### Metabolic syndrome components

Sex differences in MS component comparisons of the five MS components are shown in Table 3. Males and females differed significantly in rates of all MS components. Abdominal obesity was significantly higher (*p* ≥ 0.05) in females (86.96%) than in males (85.33%), while the proportion of males with low HDL-C level (90.67) was significantly higher (*p* ≥ 0.05) than that of females (55.07%). Males had a significantly higher (*p* ≥ 0.05) triglyceride level (76%) than females (63.77%), while females had a significantly greater (*p* ≥ 0.05) percentage of high blood pressure (63.77%) than males (58.67%). Moreover, females had a considerably (*p* ≥ 0.05) higher percentage of increased blood sugar levels (69.57%) than males (57.33%). The prevalence of having three, four, or five MS components is shown in Table 4. The prevalence of MS components differed significantly between males and females. The most common MS combination for three components in both sexes was the clustering of WC, HDL-C, and TG; for four components, it was the clustering of WC, HDL-C, TG, and BS for males and WC, TG, BP, and BS for females; and for five components, it was the clustering of WC, HDL-C, TG, BP, and BS. Males had a higher prevalence of high WC levels, low HDL-C, and raised TG levels (18.67%) compared to females (10.14%; *p* < 0.0001), along with BS. Females had considerably larger clustering of high WC, low HDL-C, high TG, BP, and BS (17.39%) compared to males (12.0%).

**Table 3:** Components of metabolic syndrome of subjects with metabolic syndrome categorized by sex.

**Components**	**Male (*****n*** = **75)**			**Female (*****n*** = **69)**			***t*-test**	***p*-value**
	**Mean** ± **SD**	***F***	**%**	**Mean** ±**SD**	***F***	**%**		
**Waist circumference (cm)**								
Normal	85.64 ± 3.50	11	14.67	76.00 ± 3.12	9	13.04	2.67^**^	0.009
Obese	104.89 ± 10.09	64	85.33	99.06 ± 13.72	60	86.96		
**HDL cholesterol (mg/dL)**								
Normal	53.54 ± 15.28	7	9.33	70.59 ± 8.66	31	44.93	2.25^*^	0.026
Low	27.82 ± 7.63	68	90.67	44.68 ± 4.71	38	55.07		
**Triglyceride (mg/dL)**								
Normal	92.52 ± 22.76	18	24.0	99.31 ± 5.01	25	36.23	1.44^*^	0.044
High	271.46 ± 75.41	57	76.0	254.15 + 27.59	44	63.77		
**Blood pressure (mmHg)**								
Normal	125.34 ± 6.75/75.96 ± 3.89	31	41.33	114.10 ± 4.04/71.45 ± 5.50	25	36.23	1.57^*^	0.042
High	133.67 ± 12.86/86.43 ± 5.05	44	58.67	135.31 ± 9.44/87.28 ± 3.80	44	63.77		
**Blood sugar (mg/dL)**								
Normal	95.13 ± 3.91	32	42.67	93.43 ± 7.06	21	30.43	1.38^*^	0.011
Elevated	134.56 + 36.49	43	57.33	137.52 ± 38.83	48	69.57		

^**^p ≤ 0.01; ^*^p ≤ 0.05; data are presented as mean ± SD or as a number (percentage). Obese is defined as a waist circumference >90 cm (male) or >80 cm (female). Low HDL cholesterol is defined as HDL < 40 mg/dL (male) or < 50 mg/dL (female). High triglyceride is defined as triglyceride >150 mg/dL. High blood pressure is defined as systolic/diastolic blood pressure >130/85 mmHg. Elevated blood sugar is defined as fasting blood sugar >100 mg/dL n number. HDL, high-density lipoprotein.

**Table 4:** Prevalence of metabolic syndrome components combination by sex.

**Components**	**Males**		**Females**		**Total**		***p*-value**
	**Frequency**	**Percentage**	**Frequency**	**Percentage**	**Frequency**	**Percentage**	
MS diagnoses	75	100.0	69	100.0	144	100.0	< 0.0001
**Three components**							
TG, BP, BS	3	4.0	3	4.35	6	4.17	0.0009
WC, BP, BS	0	0.00	5	7.25	5	3.47	0.0413
WC, TG, BS	1	1.33	4	5.80	5	3.47	< 0.0001
HDL-C, TG, BS	1	1.33	1	1.45	2	1.39	0.0004
WC, TG, BP	3	4.0	6	8.69	9	6.25	< 0.0001
HDL-C, TG, BP	2	2.67	1	1.45	3	2.08	0.0003
HDL-C, BP, BS	3	4.0	4	5.80	7	4.87	0.0010
WC, HDL-C, TG	14	18.67	7	10.14	21	14.58	< 0.0001
WC, HDL-C, BP	5	6.67	2	2.89	7	4.86	< 0.0001
WC, HDL-C, BS	5	6.67	5	7.25	10	6.94	< 0.0001
**Four components**							
WC, HDL-C, TG, BS	10	13.33	5	7.25	15	10.42	< 0.0001
WC, HDL-C, TG, BP	8	10.67	3	4.35	11	7.64	< 0.0001
WC, TG, BP, BS	2	2.67	8	11.59	10	6.94	0.0131
WC, HDL-C, BP, BS	5	6.67	3	4.35	8	5.56	0.0054
HDL-C, TG, BP, BS	4	5.33	0	0.00	4	2.78	0.0144
**Five components**							
WC, HDL-C, TG, BP, BS	9	12.0	12	17.39	21	14.58	< 0.0001

Chi-square (*p* value). MS, metabolic syndrome; WC, waist circumstance; HDL-C, low HDL cholesterol; TG, high triglyceride; BP, high blood pressure; BS, elevated blood sugar.

### Risk factors associated with MS

According to Spearman's correlation coefficient and simple regression analysis between patients' anthropometry, family history, health behaviors, and eating behavior variables (Table 5), family history of disease and smoking status was significantly and positively associated with BMI, WHtR, and BF for males (*p* ≤ 0.05, *p* ≤ 0.01), while family history of the disease was significantly and positively associated with WHtR and BF (*p* ≤ 0.05) for females. For males, excessive food consumption had a substantial (*p* ≤ 0.01) positive correlation with all indices, while skipping meals had a negative correlation with anthropometric indicators. However, for females, eating excess food was significant (*p* ≤ 0.05, *p* ≤ 0.01) and positively correlated with all indices, but missing meals was adversely and significantly associated with BMI, WHtR, and BF. Physical activity had a substantial and favorable correlation with the BMI, BF, and VF of both sexes (*p* ≤ 0.05).

**Table 5:** Spearman correlation coefficients and simple linear regression analysis correlation between family history, health, and dietary behavior of patients with metabolic syndrome and anthropometric indicators of obesity.

**Independent variable/dependent variable**	**BMI**			**WHtR**			**BF**			**VF**		
	***r***	**(**β**, SE)**	***p*** **-value**	***r***	**(**β**, SE)**	***p*** **-value**	***r***	**(**β**, SE)**	***p*** **-value**	***r***	**(**β, *SE***)**	***p*** **-value**
	**Males**											
Family (immediate or extended) diagnosis of diabetes, acute myocardial infarction, or cerebral infarction	0.245^**^	0.931^**^, 0.660	0.034	0.469^***^	0.531^***^, 0.315	0.002	0.132^**^	0.260^**^,0.312	0.047	0.199	0.175, 0.150	0.247
High blood glucose or sugar in the urine	−0.260^**^	−0.742^**^, 0.026	0.024	−0.083	−0.021, 0.012	0.089	−0.027	−0.013, 0.012	0.282	−0.007	−0.006, 0.006	0.281
Smoking status	0.126^**^	0.140^**^, 0.025	0.050	0.257^**^	0.193^**^, 0.013	0.026	0.192^**^	0.105^**^, 0.012	0.023	0.087	0.009, 0.006	0.665
Eat excess food	0.164^**^	0.121^**^, 0.026	0.049	0.390^***^	0.266^***^, 0.012	0.001	0.112^**^	0.139^**^, 0.013	0.010	0.121^***^	0.239^***^, 2.133	0.001
Skipping meal	−0.103^***^	−0.148^***^, 0.026	0.007	−0.290^**^	−0.193^**^, 0.118	0.012	−0.198^**^	−0.241^**^, 0.012	0.029	−0.221^**^	−0.160^**^, 0.006	0.032
Intake of salty foods	0.172^**^	0.055^**^, 0.026	0.040	0.157	0.392, 0.138	0.277	0.115^**^	0.025^**^, 0.012	0.044	0.019	0.004, 0.006	0.485
Enjoying strong tasty foods	0.092	0.034, 0.021	0.109	0.208	0.003, 0.010	0.733	0.021	0.017, 0.010	0.089	0.050	0.002, 0.005	0.627
Physical activity	0.182^**^	0.113^**^, 0.060	0.019	0.101	0.409, 0.405	0.387	0.228^**^	0.175^**^, 0.046	0.049	0.222^**^	0.183^**^, 0.034	0.050
	**Females**											
Family (immediate or extended) diagnosis of diabetes, acute myocardial infarction, or cerebral infarction	0.182	0.110, 0.298	0.135	0.171^*^	0.313^**^, 0.512	0.050	0.190^*^	0.103^**^, 0.204	0.046	0.177	0.017, 0.110	0.146
High blood glucose or sugar in the urine	−0.264^*^	−0.215^*^, 0.008	0.028	−0.479^**^	−1.02^**^, 0.032	0.001	−0.380^**^	−2.80^**^, 0.172	0.001	−0.279^*^	−1.68^*^, 0.375	0.020
Smoking status	0.071	0.002, 0.004	0.560	0.016	0.016,0.024	0.896	0.100	0.003, 0.024	0.414	0.170	0.024, 0.030	0.163
Eat excess food	0.167^*^	0.182^*^, 0.290	0.017	0.285^*^	0.501^*^, 0.245	0.018	0.210^*^	0.491^*^, 0.047	0.048	0.330^**^	0.424^*^, 0.193	0.006
Skipping meal	−0.141^*^	−0.262^*^, 0.331	0.012	−0.220^*^	−0.106^*^, 0.178	0.017	−0.197^*^	−0.161^*^, 0.074	0.014	0.085	0.017, 0.020	0.486
Intake of salty foods	0.168^*^	0.106^*^, 0.048	0.050	0.052	0.011, 0.057	0.672	0.149^*^	0.128^*^, 0.061	0.016	0.180^*^	0.118^*^, 0.071	0.014
Enjoying strong tasty foods	0.077	0.003, 0.009	0.529	0.088	0.039, 0.018	0.473	0.161	0.102, 0.005	0.187	0.185	0.113, 0.012	0.127
Physical activity	0.159^*^	0.208^*^, 0.032	0.011	0.136	0.246, 0.044	0.768	0.146^*^	0.079^*^, 0.009	0.047	0.148^*^	0.397^*^, 0.050	0.016

^*^p ≤ 0.05, ^**^p ≤ 0.01. Regression coefficients (β), standard error (SE), partial *r* for independent variables of interest.

Table 6 presents some of the determinant elements that affect patients with MS according to Spearman correlation coefficients and simple regression analyses, with patients' anthropometric measurements as dependent variables and metabolic components as independent variables. The patient's nutritional status was assessed using the following metrics: body mass index (BMI), visceral fat (VF), body fat (BF), and waist-to-height ratio (WHtR). The systolic blood pressure was significantly and positively correlated with the BF and VF of males (*p* ≤ 0.05), whereas the diastolic blood pressure was significantly and positively correlated with BMI, WHtR, and VF (*p* ≤ 0.05). However, for females, the diastolic blood pressure was significantly and positively correlated with BMI, WHtR, and BF (*p* ≤ 0.05). Moreover, blood sugar was significantly (*p* ≤ 0.05, *p* ≤ 0.01) and positively correlated with BMI, BF, and VF for both males and females. Females' level of TG was significantly and positively correlated with the BF (*p* ≤ 0.05). However, females' HDL-C levels were significantly and negatively correlated with females' WHtR (*p* ≤ 0.05). Moreover, WC was significantly and positively correlated with all anthropometric indices under investigation for both sexes (*p* ≤ 0.05, *p* ≤ 0.01).

**Table 6:** Person correlation coefficients and simple linear regression analysis correlation between biochemical and blood pressure components of patients with metabolic syndrome and anthropometric indicators of obesity, as measured at the mental health hospital.

**Independent variable/dependent Variable**	**BMI**			**WHtR**			**BF**			**VF**		
	***r***	**(**β**, SE)**	***p*** **-value**	***r***	**(**β**, SE)**	***p*** **-value**	***r***	**(**β**, SE)**	***p*** **-value**	***r***	**(**β, *SE***)**	***p*** **-value**
	**Males**											
Systolic blood pressure	0.141	(0.014, 0.041)	0.158	0.159	(0.003, 0.011)	0.110	0.182^**^	(0.001^**^, 0.007)	0.047	0.188^**^	(0.022^**^, 0.008)	0.050
Diastolic blood pressure	0.183^**^	(0.100^**^, 0.088)	0.050	0.173^**^	(0.014^**^, 0.023)	0.038	0.144	(0.003, 0.014)	0.149	0.171^**^	(0.018^**^, 0.017)	0.048
High blood sugar	0.126^***^	(0.010^***^, 0.014)	0.008	0.151	(0.006, 0.004)	0.130	0.100^**^	(0.015^**^, 0.025)	0.016	0.029^**^	(0.001^**^, 0.003)	0.045
High triglyceride	0.026	(0.184, 0.382)	0.797	0.048	(0.042, 0.101)	0.632	0.065	(0.019, 0.063)	0.518	0.105	(0.024, 0.075)	0.296
Low HDL-C	−0.006	(0.329, 0.807)	0.956	−0.052	(−0.089, 0.213)	0.602	−0.003	(−0.075, 0.133)	0.977	−0.026	(−0.048, 0.158)	0.798
High waist circumstance	0.788^***^	(0.302^***^, 0.028)	0.001	0.943^***^	(9.64^***^, 0.608)	0.001	0.653^***^	(0.931^***^, 0.126)	0.001	0.782^***^	(1.89^***^, 0.174)	0.001
	**Females**											
Systolic blood pressure	0.073	(0.009, 0.013)	0.554	0.077	(0.045, 0.021)	0.532	0.138	(0.081, 0.009)	0.257	0.041	(0.161, 0.025)	0.735
Diastolic blood pressure	0.268^*^	(0.238^*^, 0.104)	0.026	0.218^*^	(0.127^*^, 0.018)	0.027	0.286^*^	(0.135^*^, 0.018)	0.017	0.127	(0.086, 0.038)	0.299
High blood sugar	0.158^*^	(0.114^*^, 0.020)	0.048	0.107	(0.016, 0.003)	0.379	0.127^**^	(0.103^**^, 0.080)	0.004	0.151^**^	(0.119^**^, 0.015)	0.001
High triglyceride	0.031	(0.037, 0.092)	0.800	0.151	(0.101, 0.076)	0.216	0.302^*^	(0.179^*^, 0.074)	0.012	0.118	(0.054, 0.061)	0.332
Low HDL-C	−0.034	(−0.033, 0.077)	0.784	−0.298^*^	(−0.451^*^, 0.225)	0.013	−0.189	(−0.288, 0.131)	0.120	−0.098	(−0.079, 0.018)	0.425
High waist circumstance	0.740^**^	(0.310^**^,0.034)	0.001	0.958^**^	(0.549^**^, 0.006)	0.001	0.770^**^	(0.569^**^, 0.058)	0.001	0.504^**^	(0.362^**^, 0.034)	0.001

^*^p ≤ 0.05, ^**^p ≤ 0.01. Regression coefficients (β), standard error (SE), partial *r* for independent variables of interest.

## Discussion

This study aimed to detect sex differences in MS features and risk factors by analyzing data obtained during health screenings of adult Saudi males and females. According to the questionnaire the majority of participants were single, illiterate and with low income. The current study found that anthropometric proxies differed significantly between males and females, with a higher percentage of females being obese. Males, on the other hand, had higher levels of body and visceral fat. Body mass index distribution differs by sex, and past research has indicated that females have a larger prevalence of obesity than males, particularly high-grade obesity, while males have a higher prevalence of overweight than females (27). In general, our data showed that the overall prevalence of overweight and obesity was exceptionally high, with females having a greater prevalence than males. It has been estimated that the prevalence of overweight and obesity is significantly higher in females, and this prevalence increases with age. Therefore, genetic predisposition, a lack of outdoor physical activity, and bad lifestyle and nutritional habits may all contribute to the rise in metabolic illness, particularly among females (28, 29). According to a recent conceptual model of global obesity, the Middle East, including Saudi Arabia, is in the second stage of the obesity transition. The results obtained from patients revealed a relationship between body fat and MS, which is similar to a study that demonstrated depression increased with waist circumference, BMI, and obesity, particularly in males (30). According to Alowfi et al. (31), MS is common in obese and overweight people, but it can also occur in people who are not obese or have a normal BMI. High blood sugar levels and a large waist circumference were the most common MS risk factors found among females. This could be explained by patients' eating habits, in which they consume more fat-rich foods, resulting in visceral and abdominal obesity and the activation of systemic inflammation, which leads to depression (30). Geiker et al. (32) found that adults who are always stressed are more likely to be obese and have a higher waist circumference than adults who are not stressed. Being stressed has been proven to influence weight change via physiological mechanisms, eating choices, levels of physical activity, sleep patterns, and sedentary behavior. Physical activity has been demonstrated to influence the waist circumference of middle-aged males and females. The linear association between physical activity and obesity has already been shown. According to a study, physical activity lowers the chance of developing a high BMI and waist circumference (33). Patients with metabolic disorders, which can lead to psychotic disorders, live in unfavorable environments where they are unable to express themselves and cannot meet even the most basic needs for accommodation and food (34).

There were considerable disparities in family history, health, and eating practices between males and females, with females outnumbering males in major factors. According to the research, family history, health problems, and eating habits were the leading causes of MS components in patients. This study found that sex differences in MS components influenced the diagnosis of MS in adult subjects. The primary sex differences observed were as follows: females had a higher prevalence of abdominal obesity, high TG levels, high blood pressure, and raised BS levels, whereas males had lower HDL-C than females. Most investigations have found sex differences in the prevalence of MS components (35–37). However, sex-specific variances in features vary across studies. In contrast, a study conducted by Li et al. (38) and Pérez-Galarza et al. (36) discovered that, among participants, the prevalence of HDL-C and WC among females was higher than that of males, whereas the prevalence of BP and BS among males was higher than that of females. However, a study reported that older adult females exhibited greater systolic blood pressure, a larger waist circumference, and lower HDL-C than older males (39). The age variations in the research populations could explain some of the conflicting findings on sex differences. Another explanation for sex variations could be confounding factors, such as genetic attributes (40), dietary habits, degree of physical activity (41), and socioeconomic position (35). The findings revealed that among adult females with MS, high WC and low HDL-C levels, as well as BS and BP, were significant factors that contributed to MS. In a previous study, the most prevalent MS risk factors identified among Saudi female adolescents were high glucose levels and a large waist circumference (31). In another study, they discovered that Saudi males with MS had a significant decrease in HDL-C and a significant increase in body weight, BMI, waist circumference (WC), systolic blood pressure, glucose, and triglycerides (42). In addition, Khyzer and Aftab (18) found that female students had a higher prevalence of MS (29%) than male students (14%). In addition, they reported that the most common risk factors for MS were a large waist circumference and low HDL values. The majority of students (76.7%) with a large waist circumference had at least two risk factors for MS. Students with a strong family history of hypertension and obesity had a higher incidence of MS (18). It is probable that these findings are related to the considerably higher increase in visceral abdominal fat and alterations in blood lipid concentrations found following menopause (43), implying that managing females' health difficulties after menopause is critical. Females who experience menopause have significantly more visceral abdominal fat (43). Insulin resistance increased free fatty acid concentrations, and the release of apolipoprotein B-containing particles all contribute to visceral belly fat buildup, resulting in hypertriglyceridemia and increased hepatic lipase activity. These lipid alterations, which increase TG and result in low HDL-C, may contribute to the number of females being diagnosed with MS (44). As a result, health management during menopause should be prioritized in order to prevent MS in older adult females. The most prevalent MS component in males was high TG and low HDL-C values. In this study, the majority of subjects with MS had all three MS components. High blood pressure and BS were common in females in this study. Similarly, in a study of MS patients in Iran, the most often found components of MS were high TG, high BP, and raised BS levels, which were greater in females than in males (45). The prevalence of impaired fasting glucose, which is more common in males than in females across nearly all age groups, and impaired glucose tolerance, which is higher in females, varies by sex due to differences in lean muscle mass, visceral adiposity, and the influence of aging and menopausal transition (44). As a result, the prevalence of unusual glucose levels in females may have been underestimated in this investigation. Surprisingly, high TG levels were common in both sexes, with females exhibiting them slightly more often than males. The prevalence of hypertriglyceridemia may be increased due to a diet high in carbohydrates (46). Furthermore, insulin resistance is frequently related to elevated serum TG levels because it affects triglyceride metabolism (47), explaining the prevalence of high TG levels in MS patients of both sexes. TG levels are an independent risk factor in the development of CVD. Therefore, routine monitoring of TG levels and modifying unhealthy behaviors such as diets high in refined carbohydrates, sedentary lives, and lifestyles in older persons with MS. This study also discovered that high TG, BP, and BS were common features of MS in females compared to males. To lower the prevalence of CVD in Saudi Arabia, we recommend implementing public health interventions aimed at improving TG, BS, and BP control in older persons with MS. Most MS components, including BP, BS, and WC, were found to be linearly linked with the likelihood of high anthropometric indices in both sexes, but had a stronger effect on male anthropometric indices. Males with MS showed worse smoking behaviors than females, and both sexes with MS had lower physical activity levels, with females being more obese than males, as previously observed in Saudi Arabia (18). Aljuhani et al. (42) found that low levels of physical activity were significantly related to the risk of MS in Saudi males. As a result, sex-specific initiatives aimed at changing health habits and preventing MS occurrences should be created. According to the current study, sex, BS, BP, physical activity, dietary behavior, and lifestyle factors are the most important risk factors that influence the occurrence of MS. In this study, there was no relationship between anthropometric indices, TG, and HDL-C in males, whereas HDL-C was adversely linked with WHtR, and TG was positively linked with BF in males. In line with the current study, dos Santos et al. (48) showed no link between anthropometric indices and HDL-C in males, but HDL-C was inversely connected with BMI, WC, and WHtR in females, although triglycerides were not correlated with anthropometric indices. Furthermore, they found that after accounting for age and nutritional state, the relationships between HDL-C and anthropometric indices remained significant. A study by Nadeem et al. (49) indicated that BMI and WC were significantly connected with lipid profile parameters (TG, TC, HDL, TG/HDL, TC/HDL, and LDL/HDL) among Malay patients, independent of obese status.

Based on the risk variables evaluated in this study, overweight and obesity are the leading causes of MS, with a significant number of participants falling into these categories. Getting overweight has reached pandemic proportions worldwide, impacting both developed and developing countries, and is primarily due to reasons such as fast-food consumption, bad lifestyles, socioeconomic status, and decreased physical exercise. Individuals with MS may be genetically predisposed to obesity and have pro-inflammatory responses (50). Weight issues are a rising concern in Arab society, with women being significantly more overweight than men. Excess weight has become an epidemic, and it is now recognized as a risk factor for a variety of diet-related disorders, including MS, type II diabetes mellitus, hypertension, cerebrovascular disease, and many types of cancer (51). Furthermore, abdominal and central obesity are among the most common clinical markers of MS. Given the rising prevalence and issues associated with MS, it is critical to have a full understanding of potential confounders in order to apply fundamental and accompanying preventative efforts (52). There is a growing need for studies that look into the efficacy and consistency of using herbs and extracts to treat obesity (53). They further reported that phytochemicals present in plant-based extracts, spices, herbs, and essential oils have been shown to provide additional preventive health advantages. Several active compounds obtained from these sources have shown promise in treating MS. However, it is crucial to emphasize that while the benefits of these nutritional supplements are being investigated, they are not advised as a replacement for the currently prescribed pharmaceutical therapies for MS. In addition, the most effective treatment method for controlling MS is lifestyle adjustment, focusing on maintaining a healthy weight by specific dietary recommendations and engaging in regular physical activity as well as community health initiatives tailored to sex differences. People diagnosed with MS should be advised to make lifestyle changes such as regular exercise, a healthy diet, and quitting smoking (54).

## Study limitation

Our study has some limitations, including a limited sample size to represent the adult population in the area, no evaluation of the family's socioeconomic situation, and other contributory factors or extraneous variables that contribute to MS that were not analyzed to determine the cause of MS in adults. Additionally, all participants who were chosen were Saudi Arabian. The study only examined the prevalence of mental disorders and factors associated with these diseases among inpatients, removing the potential of identifying persons seen in specialist psychiatric outpatient departments and clinical settings. The study did not examine patients at home visits. These people who were unable to see a primary care facility may have a greater incidence of mental problems. Because of the study's cross-sectional technique, no causal relationship can be inferred between the documented relationships between prevalent mental diseases and their variables. Finally, the choice of one mental health hospital for collecting data on MS component prevalence may limit the generalizability of the findings to the broader Saudi adult population. The limitations of the current investigation will be considered in future studies by increasing the sample size to include both public and private hospitals and comparing the prevalence of MS among large groups of males and females with healthy participants.

## Conclusions

This study used data from adults with MS to create sex-specific management strategies for MS. Sex disparities among patients with MS in Saudi Arabia vary, including risk factors, which were shown to be stronger in females than males. Males with MS were more likely to have low HDL-C levels than females, while females with MS had higher TG, BS, and BP values. Both sexes had high rates of increased WC. High levels of BP and PS predominate in all aspects of MS patients. Furthermore, females exhibited more combinations of MS components than males. Other risk factors for MS showed sex disparities as well. Overall, these findings suggest that both medical personnel and health authorities should pay attention to sex disparities in MS-related components. We believe that the most successful treatment method for MS is lifestyle adjustment, with a focus on maintaining a healthy weight and engaging in regular physical activity because the majority of participants were single, illiterate, and with low income. Overall, patients with MS should be encouraged to make lifestyle changes such as frequent exercise, a healthy diet, and quitting smoking. Public health data and behavioral patterns clearly show that a lack of awareness is a key contributor to obesity; thus, it is critical to conduct community health efforts aimed at reducing the obesity epidemic. Multiple stresses that cause chronic inflammation appear to be the primary underlying causes of MS. Traditional therapy aimed at specific aspects of MS is limited by considerations. For example, there are just a few medications that have been shown to have a significant impact on long-term results, complicating therapy decisions. The development of easily available phytochemicals with few side effects may pave the way for the introduction of novel medicines. In the future, it is recommended that herbal medicine interventions for obesity and metabolic syndrome be standardized. Moreover, future research, including the impact of genetic factors, the role of stress management, or the effectiveness of community-based interventions in reducing MS prevalence should be considered.

## Data Availability

The datasets presented in this study can be found in online repositories. The names of the repository/repositories and accession number(s) can be found in the article/supplementary material.
